# A computational platform for the virtual unfolding of Herculaneum Papyri

**DOI:** 10.1038/s41598-020-80458-z

**Published:** 2021-01-18

**Authors:** Sara Stabile, Francesca Palermo, Inna Bukreeva, Daniela Mele, Vincenzo Formoso, Roberto Bartolino, Alessia Cedola

**Affiliations:** 1 Dipartimento di Fisica, Università della Calabria, 87036 Arcavacata di Rende, CS Italy; 2 Institute of Nanotechnology - CNR, Cosenza Unit, 87036 Arcavacata di Rende, CS Italy; 3 Institute of Nanotechnology - CNR, Rome Unit, Piazzale Aldo Moro 5, 00195 Rome, Italy; 4 P.N. Lebedev Physical Institute, RAS, Leninskiy pr., 53, 119991 Moscow, Russian Federation; 5 Dipartimento Scienze della Terra e Geoambientali, Università degli Studi di Bari “Aldo Moro”, Bari, Italy

**Keywords:** Applied physics, Imaging techniques, Computational methods, Computational science, Computer science, Software

## Abstract

Ancient Herculaneum papyrus scrolls, hopelessly charred in the 79 A.D. Vesuvius eruption, contain valuable writings of the Greek philosophers of the day, including works of the Epicurean Philodemus. X-ray phase contrast tomography has recently begun unlocking their secrets. However, only small portions of the text hidden inside the scroll have been recover. One of the challenging tasks in Herculaneum papyri investigation is their virtual unfolding because of their highly complicated structure and three-dimensional arrangement. Although this procedure is feasible, problems in segmentation and flattening hinder the unrolling of a large portion of papyrus. We propose a computational platform for the virtual unfolding procedure, and we show the results of its application on two Herculaneum papyrus fragments. This work paves the way to a comprehensive survey and to further interpretation of larger portions of text hidden inside the carbonized Herculaneum papyri.

## Introduction

The Herculaneum papyri were discovered in 1752. More than two and a half centuries have passed since this extraordinary finding brought to light the unique treasure of the ancient library of *Villa dei Papiri*. This is the only great ancient library that remained fundamentally intact, which contains many precious texts not handed down by Medieval manuscript tradition, as it was revealed by the papyri mechanically unrolled (or partially unrolled) in the past. In this light, reading the papyri still rolled up or the fragments, made up of several layers stuck together, could disclose unpublished ancient texts.

Carbonization, due to the Vesuvian catastrophe in 79 d.C., and the natural effects of time on organic substances (humidity and considerable pressure) during Seventeenth centuries, have contributed to the artefacts’ degradation. On the one hand, the carbonization of the papyri allowed their conservation, on the other it made them similar to pieces of burnt wood, very fragile and extremely difficult to unroll without losing large portions of the scroll.

A considerable number of rolls was destroyed or damaged within the first years after their finding: many scrolls had been thrown away as mere charcoal; others were split into two parts longitudinally to discover their contents, with irreversible effect on their integrity. Finally, after 1753, Father Antonio Piaggio invented a machine for unrolling the manuscripts, based on the movement of a screw which gradually spread out the rolls on a strip of pig or sheep bladder. Unfortunately, even under the best conditions, much of the text had been lost during this process: few unrolled parts of papyrus gave a considerable number of continuous and legible columns without large damages. Modern conservation principles impose to preserve the integrity of historical documents, and any attempt to open them must be avoided if there is a risk of losing the information they contain, also at the cost of leaving them untouched for an indefinite period. The poor conditions in which the papyri were found make them too fragile for any effort of manual opening. Therefore, the priceless information contained in them is unreachable without a non-invasive approach. In this framework, non-invasive and non-destructive X-ray imaging techniques are an ideal tool for analyzing fragile artefacts without damaging them. X-ray micro computed tomography (micro-CT) provides papyrus digitization and three-dimensional visualization of the scroll interior. The first application of micro-CT on two intact Herculaneum papyri took place in 2009^1^. Data analysis showed a tortured shape and no writing traces were recovered. Afterward, Mocella et al.^2^ and Bukreeva et al.^3^ used X-ray Phase Contrast Tomography (XPCT) to study Herculaneum papyrus rolls and fragments. It has been demonstrated the potential benefit of applying this technique to trace handwriting hidden inside the scrolls.

The main challenge of handwriting search and recognition in the papyri is due to the predominant use of carbon-based black inks, which have a much lower image contrast to the papyrus substrate than pigments with metallic bases.

Gibson et al.^4^ compared different imaging techniques by using synthetic phantoms of papyri. They have shown, in particular, that the micro-CT can detect papyrus fibers and metal-based inks, but it fails to identify carbon-based inks. However, they have proved that XPCT can detect both papyrus fibrous structure and carbon-based ink writing. Recently Parker et al.^5^ implemented machine learning pipelines for carbon ink detection in micro-CT data. The methods, tested on a small Herculaneum papyrus fragment, exploit the intensity contrast and morphological differences between the ink and the substrate. The results show that the methods are promising to detect carbon ink in authentic texts.

Starting from non-invasive X-ray acquisitions, several numerical procedures have been proposed to provide a virtual unfolding of different damaged ancient historical documents and successive revealing of hidden texts^6–11^. A procedure applied in^3^ to Herculaneum papyri led to the revealing of traces compatible with letters, words, and portions of the Greek text. However, the investigated areas of the scrolls were limited to the least damaged parts of the manuscripts and to the papyrus sheet configurations that can be virtually flattened with minimal distortions.

In this paper, we present a computational platform for the virtual 3D investigation of the papyrus fragments from the collection of carbonized scrolls found in Herculaneum in 1792. Due to the critical conditions of the fragments, virtual operations on the 3D tomographic images, such as segmentation and flattening of the papyrus sheets, are very complicated and require an approach developed specifically for this particular task. We analyzed two fragments: *PHerc.110* detached from a papyrus partially unrolled in 1867, and *PHerc.1103* which was part of the *scorza*, i.e. the outermost part of the scroll, removed before unrolling the inner part with Piaggio’s machine. The *scorza* is the most damaged part of the papyrus due to the thermal shock, the pressure, and the handling after they discover in the eighteenth century. Due to the poor condition, the *scorza* has always been neglected even if it deserves an in-depth analysis: besides the handwriting, the *scorza* could reveal precious information relating to the author, the title and the length of the work, often reported at the end of the roll.

In the following sections, we present and discuss the results of the virtual unfolding of the papyrus fragments obtained with the developed computational platform. In the “Materials and methods” section, we describe in detail each step of our approach: 3D data digitization, segmentation, modelling, flattening, and texture mapping of papyrus sheets. Volumetric data were digitized by non-destructive micro-CT and XPCT imaging techniques. The segmentation procedure digitally identifies and distinguishes a single papyrus sheet within the volume. To model the sheet surface, we applied the triangular meshing, which is fast and easy to generate. The choice of the parameterization methods for sheet unfolding is a significant issue that must be taken into account to meet the requirements of minimum distortion. Once the appropriate parameterization method was identified, the final step consists of texturing, i.e. assigning the 3D color information to each point of the 2D mesh.

## Results

The main goal of the present work was to develop a computational platform to perform the virtual segmentation and flattening of a single sheet of a carbonized papyrus fragment. This is a challenging task to deal with, especially when we handle the *scorza*, which is the most damaged part of the Herculaneum scrolls.

**Figure 1:**
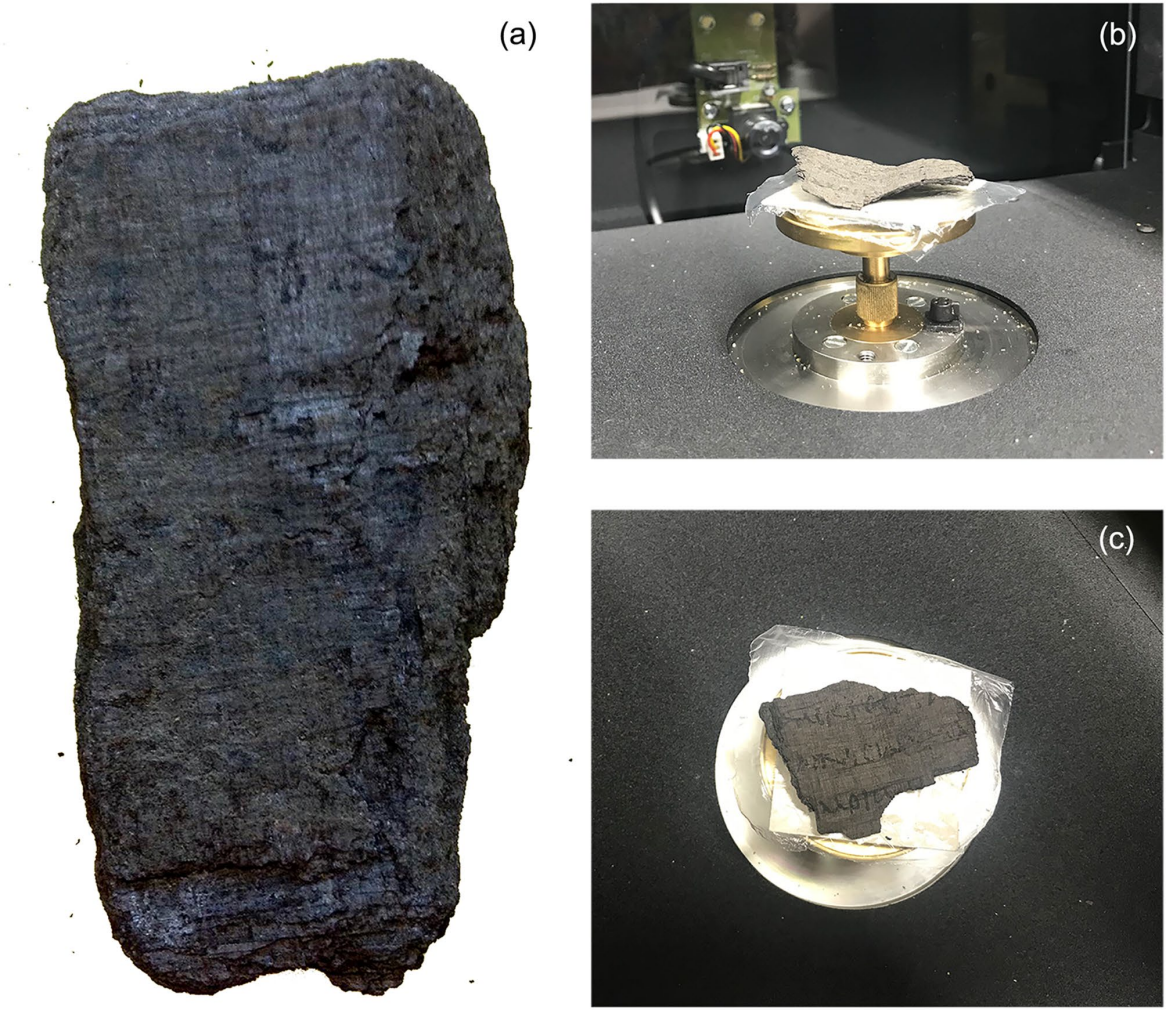
Photos of the papyrus fragments *PHerc.1103* (**a**) and *PHerc.110* (**b**,**c**). Image contrast and brightness were enhanced to better visualize the details visible to the naked eye on their external surface.

We performed XPCT and micro-CT experiments and used the experimental data to reconstruct the three-dimensional image of two historical papyrus fragments, *PHerc.1103* and *PHerc. 110*. The photographic images of the fragments *PHerc.1103* and *PHerc.110* are shown in Fig. 1a–c, respectively. *PHerc.1103* was scanned using synchrotron based XPCT, while *PHerc.110* was scanned using micro-CT with a laboratory source. The fragments, as we have indicated in the Introduction, belonged to different Herculaneum scrolls. Both fragments have an extremely compact internal structure and consist of many papyrus layers stuck together, impossible to separate manually. Only the outer surface of the fragments is accessible to the naked eye and in Fig. 1c some Greek letters can be identified.

**Figure 2:**
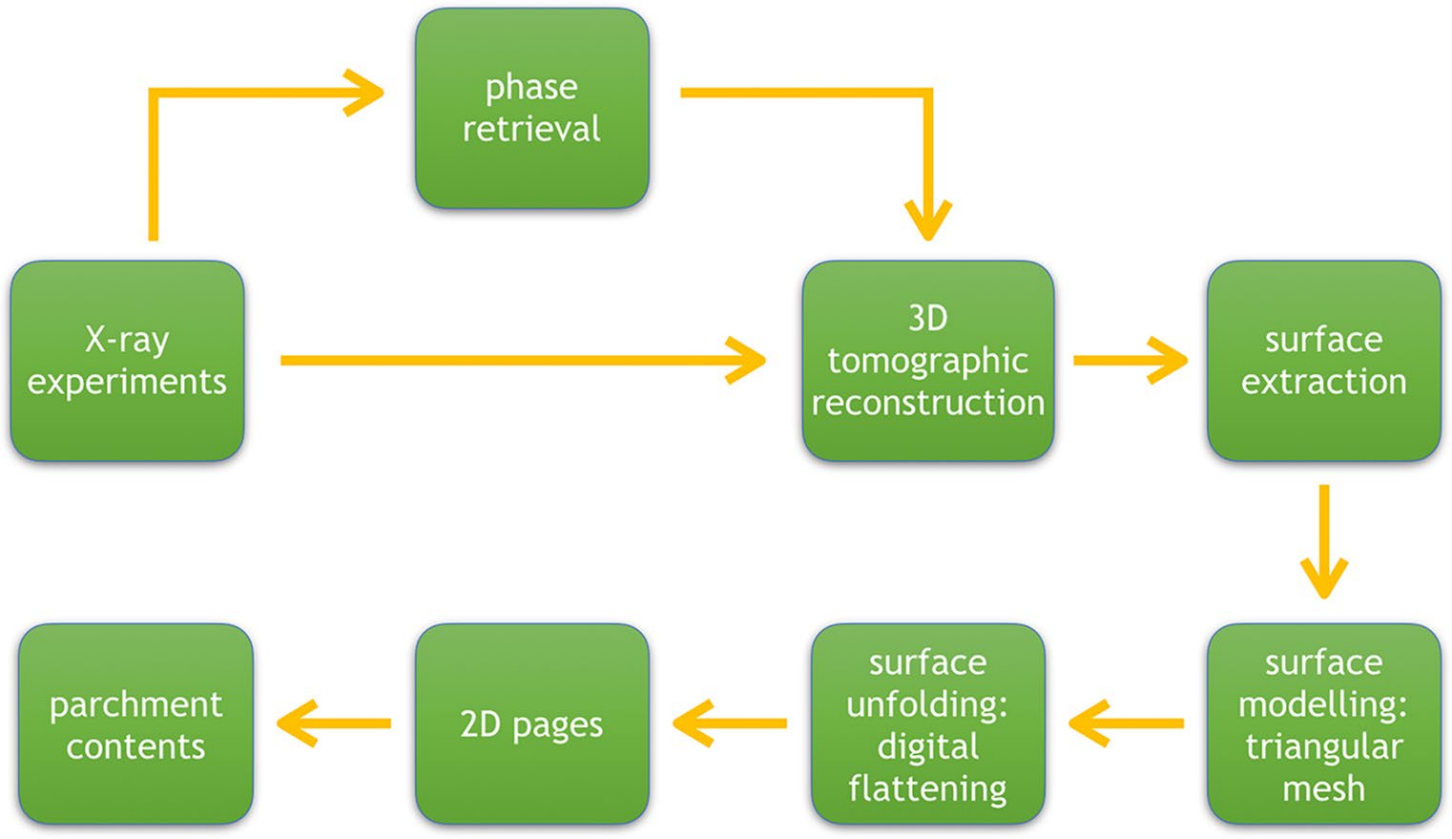
Pipeline for investigation of papyri.

Figure 2 schematizes the developed computational pipeline that includes X-ray scanning of the samples, tomographic reconstruction, extraction, modeling, and flattening of the surface (see the description of the pipeline in the “Materials and methods” section). We applied each step of the developed pipeline to both fragments.

The identification of a single papyrus sheet and the surface extraction are essential procedures for the sheet unfolding.

**Figure 3:**
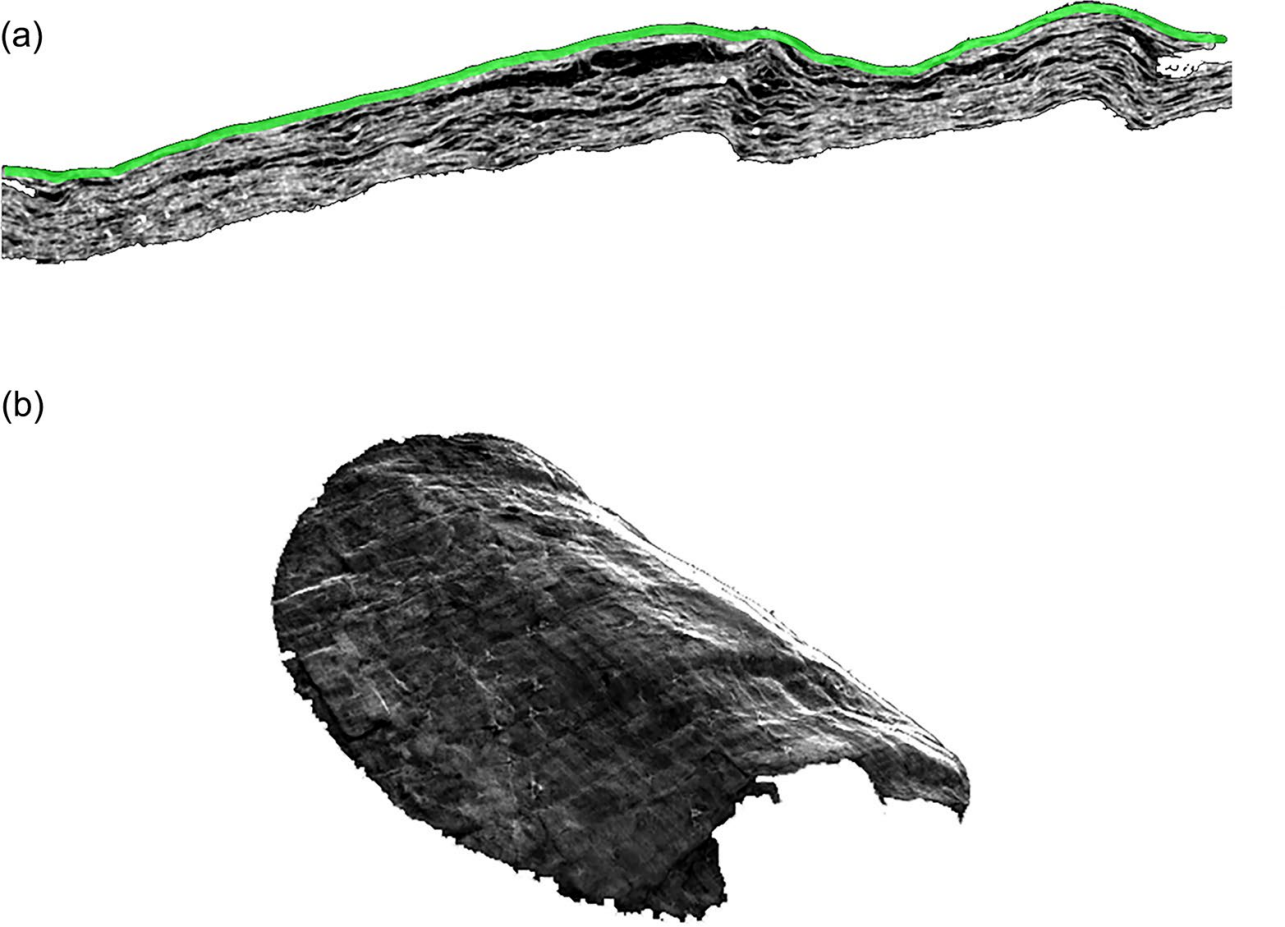
*PHerc.110* image acquired using micro-CT. (**a**) The lateral section of the tomographic volume is overlaid with a segmented sheet (highlighted with the green line). (**b**) 3D rendering of the segmented sheet.

Figure 3a shows the lateral section of *PHerc.110* obtained after the reconstruction of micro-CT data. The green line identifies the position of the outermost sheet of the fragment, and it graphically represents the sheet segmentation process. As can be observed from Fig. 3a, the fragment is composed of several layers of sheets, which appear so convoluted and deformed at a small and large scale that it is arduous to find portions of the sheet even only locally uniform. Therefore, distinguishing the sheets from each other is a complex task that we solved by a semi-manual procedure, described in the “Materials and methods” section.

3D image of a single sheet virtually extracted from the fragment *PHerc.110* is shown in Fig. 3b. The same segmentation procedure was applied to separate all the sheets of the fragment *PHerc.1103* measured with XPCT. The images of the segmented sheets of *PHerc.1103* are not present here.

**Figure 4:**
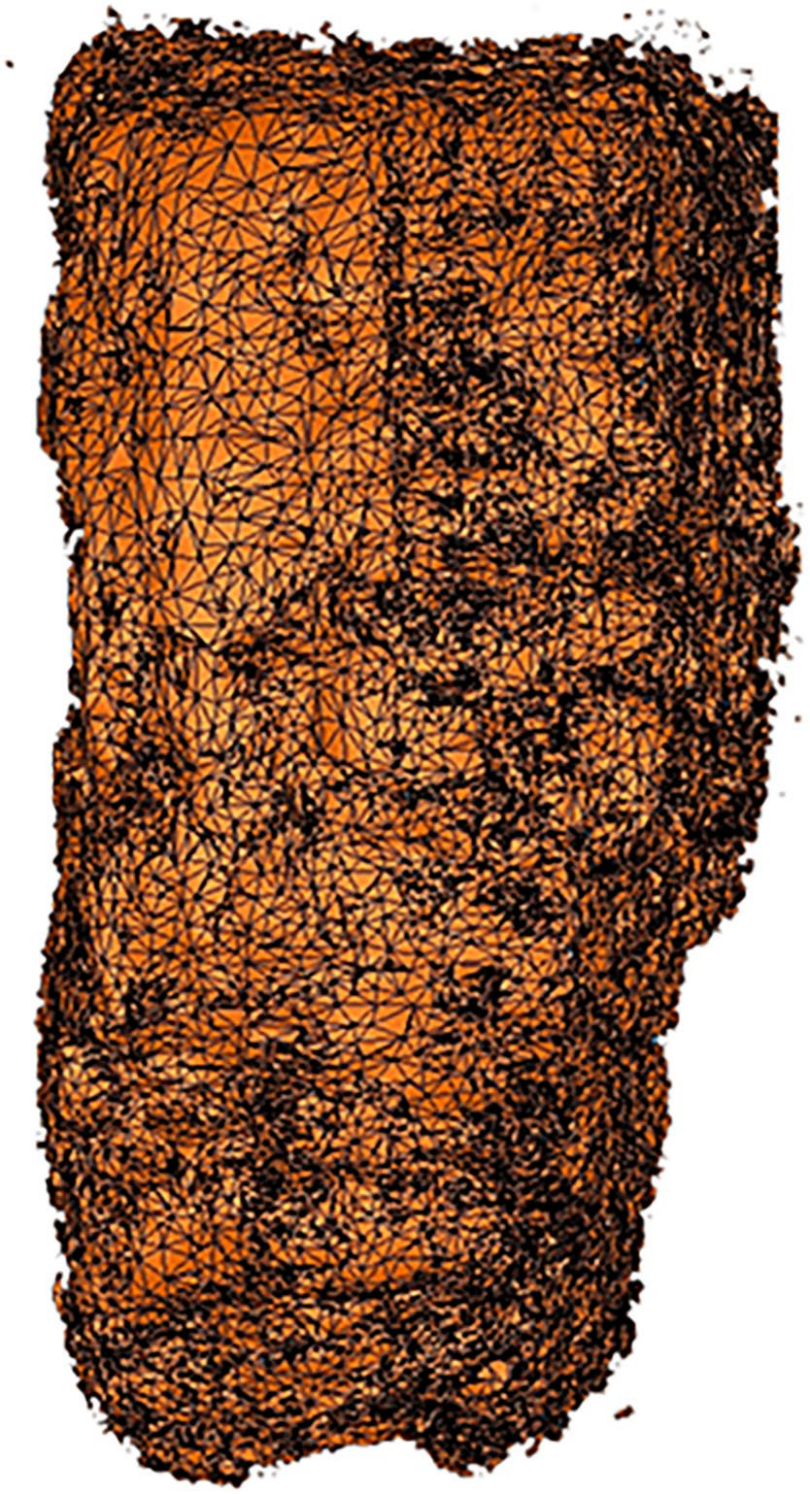
The triangular meshing of *PHerc.1103*. The figure shows a simplified representation of the mesh: the triangular density is less than in the real mesh to provide better visualization.

**Figure 5:**
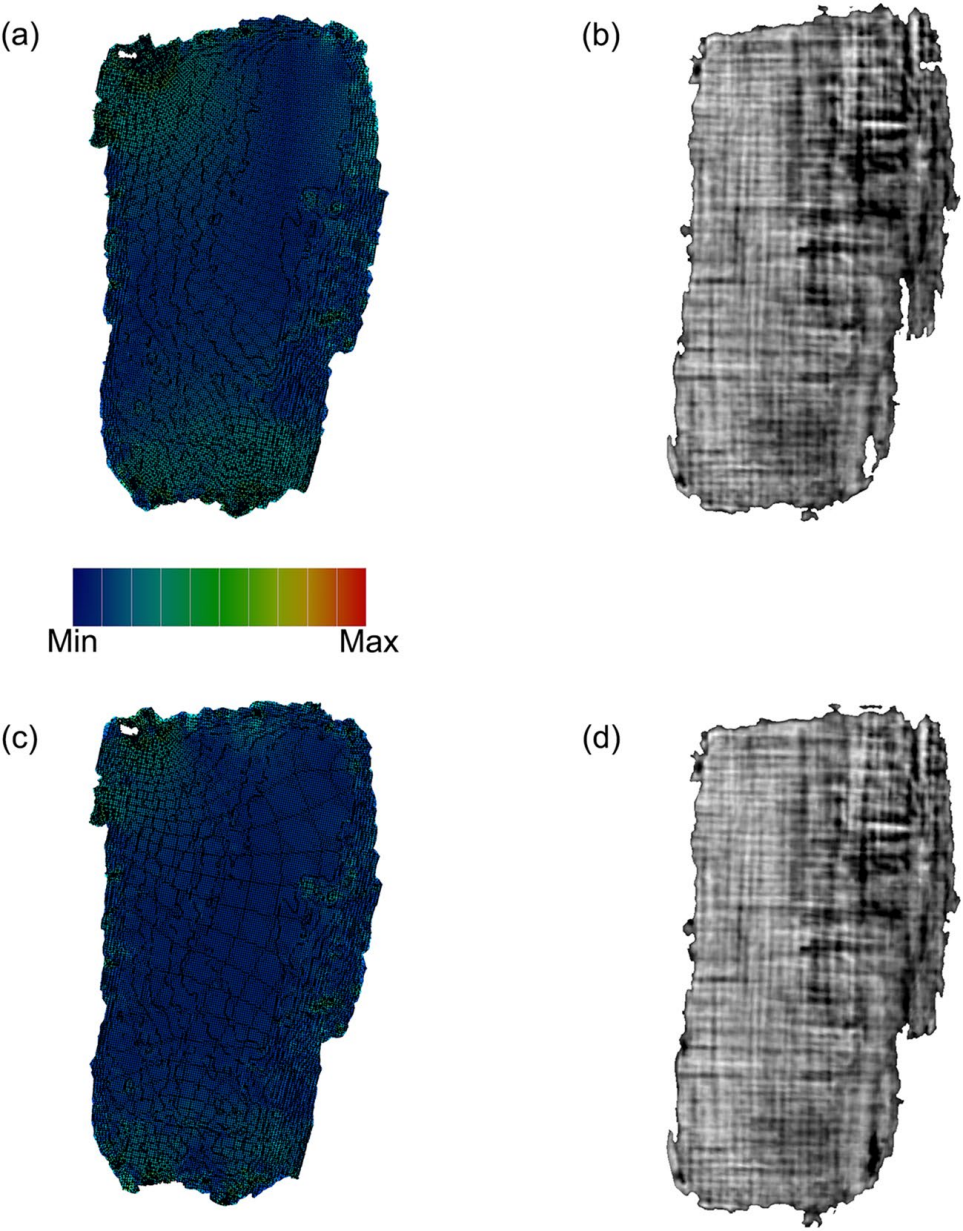
Comparison between different parameterization methods for the digital flattening of a sheet extracted from *PHerc. 1103*. 2D distribution pattern of area stretch (**a**) and 2D texture mapping (**c**) using LSCM; 2D distribution pattern of area stretch (**b**) and 2D texture mapping (**d**) using ABF. The color scale describes the degree of distortion in areas.

**Figure 6:**
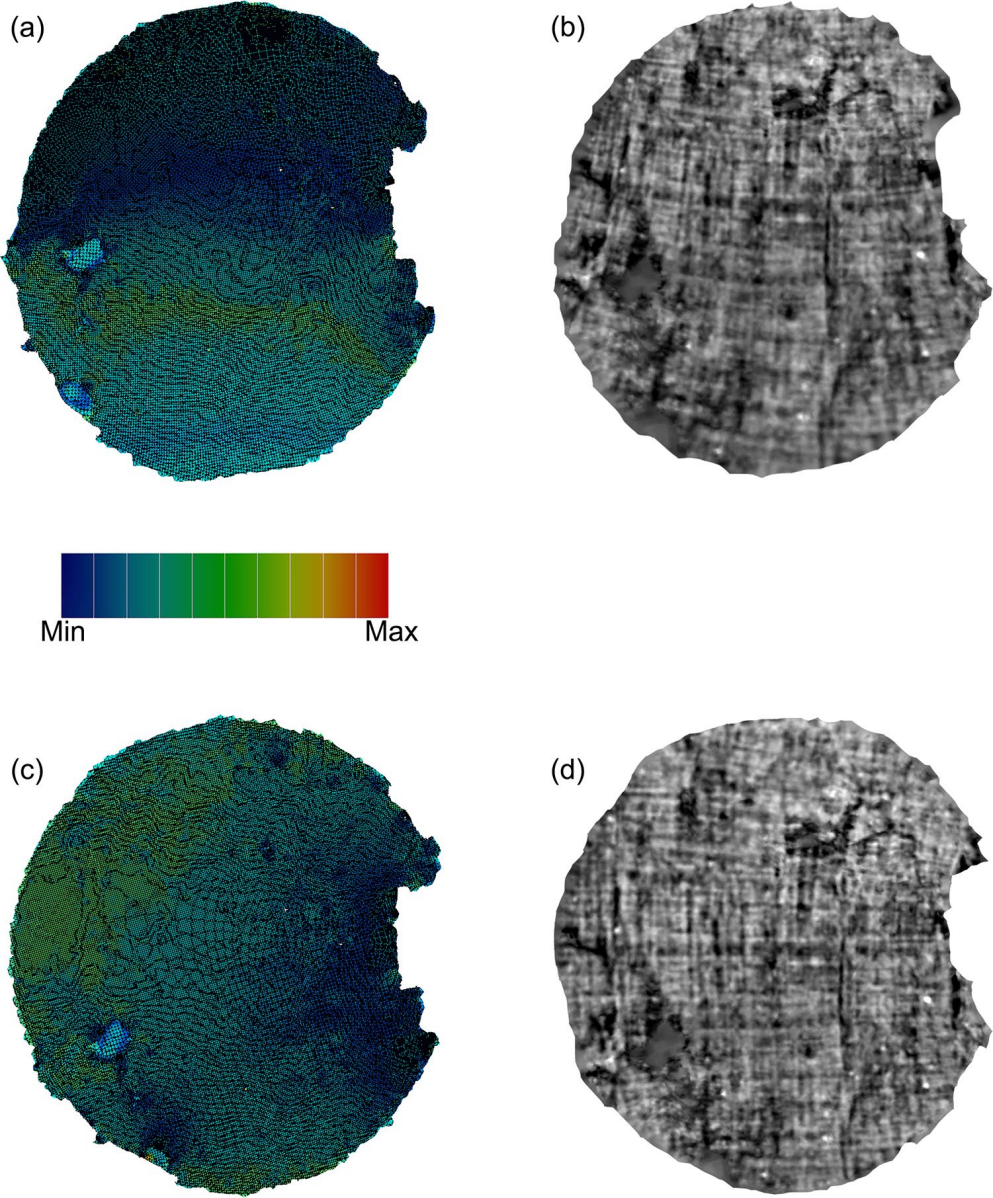
Comparison between different parameterization methods for the digital flattening of a sheet extracted from *PHerc. 110*. 2D distribution pattern of area stretch (**a**) and 2D texture mapping (**c**) using LSCM; 2D distribution pattern of area stretch (**b**) and 2D texture mapping (**d**) using ABF. The color scale describes the degree of distortion in areas.

After the surface extraction, we applied the flattening procedure. We approximated the surface using a triangular mesh, that defines the shape of objects through a set of triangles connected by their common edges. Figure 4 displays a simplified representation of the mesh (the mesh density is reduced to provide a better visualization). As the next step, we flattened the sheet; in other words, we use the surface parameterization process to map the 3D mesh onto a 2D coordinates space. Generally, this operation produces geometric deformations that are responsible for image artifacts. For this purpose, we focused on the two most popular methods, which provide parameterizations preserving angles: Least Squares Conformal Maps (LSCM)^12^ and Angle-Based Flattening (ABF)^13^. By using angle-preserving methods, local geometry is well retained, but area and length distortions are inevitably introduced. We tested both methods on each surface extracted from the tomographic volumes to find the appropriate one to process the data. Figures 5 and 6 show the results obtained with both parameterization methods for *PHerc.1103* and *PHerc. 110*, respectively. Figure 5 illustrates the results of parameterization of a surface extracted from *PHerc.1103* using LSCM (top panels) and ABF (bottom panels). In particular, Fig. 5a,c show the distortion maps, which describe the distribution of the distortion in areas; on the color scale, blue corresponds to low distortion, and red corresponds to high distortion.

By studying the distortion maps, one can assess the location of the distortions and quantify their degree. These allow to verify the reliability of the parameterization method concerning the sample in question. Figure 5a,c show that more notable distortions in *PHerc.1103*, green in color, are present near the border in the upper and lower part of the maps produced by both parameterization methods. Both LSCM and ABF parameterization method produce similar results and, therefore, we cannot assume, from Fig. 5a,c, which one is better. Figure 5b,d display the flattened textured images obtained by re-assigning the grayscale values to the figures after the parameterization. Since the fibers show a high contrast in the tomographic images obtained with both micro-CT and XPCT, we exploited the direction of the papyrus fibers as the principal criterium to estimate the accuracy of the flattening. As a single sheet was fabricated with two layers of pitch arranged perpendicularly, a well-flattened papyrus sheet should show a regular crisscross pattern, as it appears in Fig. 5b,d, which display the flattened textured images of *PHerc.1103*. Figure 5b was obtained with LSCM, and Fig. 5d with ABF parameterizations.

The flattening procedures described so far for *PHerc.1103* were then applied to the surfaces extracted from *PHerc.110*; the results for a sheet are shown in Fig. 6. Figure 6a,c shows that the distortions introduced after the flattening of *PHerc.110* are inhomogeneously distributed. The distortions due to LSCM (Fig. 6a) appear more accentuated in the lower part of the map. The result is in agreement with the textured image, shown in Fig. 6b, where a distortion appears as a bending of fibers that should be straight. The distortions in Fig. 6c, showing the map obtained from ABF, are mainly localized in the top left corner, while in the lower right corner the surface has minimal distortions. In Fig. 6d, the horizontal and vertical fibers appear straight. In this case, ABF produces better results compared with LSCM.

**Figure 7:**
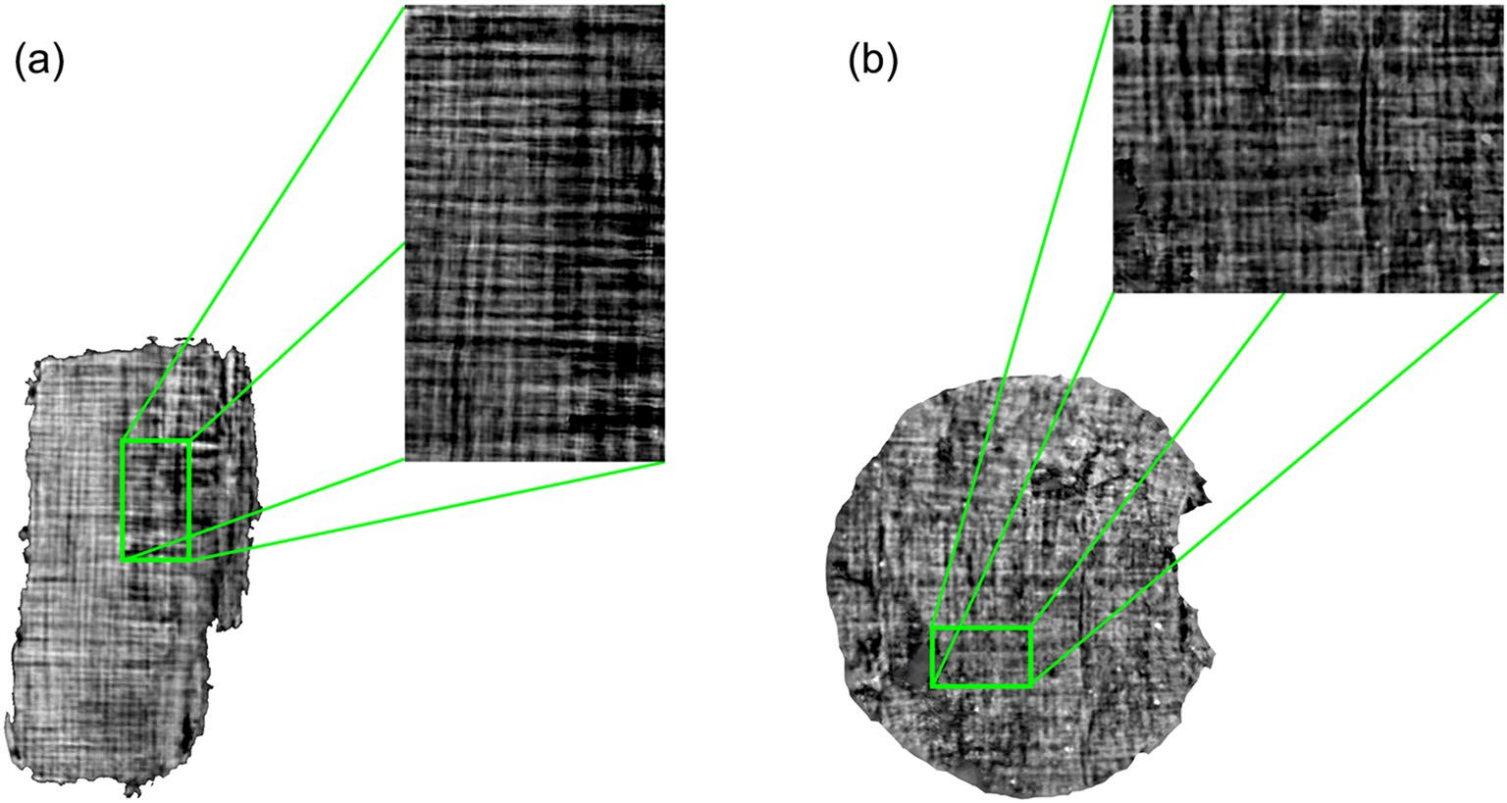
XPCT tomographic image. The figure shows two flattened sheets extracted from *PHerc. 1103* (**a**) and *PHerc. 110* (**b**). The crisscross pattern is visible in the zooms.

Figure 7 shows a zoom of the best results obtained for *PHerc.1103* (Fig. 7a) and *PHerc.110* (Fig. 7b). The segmented and flattened sheets display a regular crisscross arrangement of the fibers that proves the accuracy of the procedure.

## Discussion

We demonstrated that the developed pipeline provides an efficient virtual flattening of carbonized papyrus fragments with a highly complex internal structure. Fragments *PHerc.110* and *PHerc.1103* belonged to different scrolls partially unrolled in the past with a mechanical and destructive approach; in particular, *PHerc.1103* is a piece of the *scorza*, the outmost, and usually the most damaged part of the roll. The analysis of the *scorza* may lead to the disclosure of valuable information—the author, the title, and the length of the manuscript—as well as encourage further research to discover the content of the work.

We used non-destructive X-ray imaging techniques such as micro-CT and XPCT for the digitization of fragile artefacts without damaging them. We developed and successfully applied a computer algorithm dedicated to the virtual unfolding of Herculaneum papyrus fragments. In particular, we expect that this technique to be an appropriate virtual tool to handle the *scorza*, which is the most damaged part and, at the same time, a valuable section of the papyrus.

Both the investigated fragments of papyrus have a very compact and complicated internal structure. We used a semi-manual procedure to distinguish and virtually separate single sheets from the whole tomographic volume. An automatic procedure cannot be applied since each piece has specific geometry, fragments consist of many papyrus layers stuck together, and each layer has a very irregular structure.

After the segmentation of the papyrus sheet, we approximated its 3D surface using a mesh and applied different parameterization methods for flattening. The parameterization consists in mapping the surface from 3D to 2D coordinates space. Due to the complicated surface morphology, automatic surface parameterization does not produce acceptable levels of stretch. For this reason, the surface must be virtually cut before parameterization to reduce its complexity. The cuts have been done manually where the distortions are maxima, i.e. along the edge and the overlapped regions. A problem associated with this procedure is the appearance of visible discontinuities along with the cuts. We should, therefore, find the right compromise between distortions and cuts before proceeding with the process of parameterization.

For the procedure of sheet flattening, we compared the results obtained by using the two most popular parameterization methods, ABF and LSCM. For *PHerc.1103* (Fig. 5), both the methods produce satisfactory results, while for *PHerc.110* (Fig. 6), ABF gives a more accurate result than LSCM. The comparison between ABF and LSCM shows that there is no universal method for surface flattening, but the choice of the parameterization method depends on the condition of fragments under examination and the geometry of each sheet. Therefore, to determine the proper method, i.e. the one that better minimizes the distortion degree, it is necessary to test different algorithms and compare the results for each sample. In this regard, we stress that other parameterization methods should be tested as well, for example, the physics-based material modeling (MM) algorithm^9^.

To validate the accuracy of the flattening, we exploited the high absorption contrast in the tomographic images provided by the papyrus fibers. The comparison between the data acquired via micro-CT and XPCT confirmed that both methods produce similar results concerning fiber visibility. We proposed the use of the fibers and their direction as a guideline for the identification of the papyrus layers and the single sheet surface, and to estimate the accuracy of the flattening. A single papyrus sheet displays a crisscross pattern structure due to its production method. The sheet is composed of two layers of plant strips with parallel fibers placed at the right angle to each other. The fibers run horizontally on the *recto*, the sheet side where usually the text was written (writing surface), and vertically on the *verso*, the external layer used as support to roll the scroll^14^. The zooms in Fig. 7 show that, after the application of the flattening algorithm, the fibers of the sheets appear arranged perpendicularly. This fact allows us to state that the flattening procedure is correct. However, the surface is still uneven and irregular from macro- down to micro-scale, significantly complicating the text tracing.

The search for the text contained within the papyri is the ultimate goal of the virtual investigation. In Herculaneum papyri, the prevalent use of carbon-based black ink in writing represents one of the most challenging issues to address. Carbon-based inks have a much lower image contrast than metallic inks and cannot be detected by micro-CT. The carbonization of the papyri makes the problem more complicated since it is necessary to distinguish traces of carbon-based ink from a substrate with very similar characteristics. XPCT has shown promise to detect carbon-based ink writing^4^, thanks to its ability to provide image contrast for light materials, such as carbon. This technique can detect small variations of density that can help to distinguish the carbon ink from the carbonized papyrus sheet. However, to achieve a reliable differentiation of the text from the papyrus structure, future XPCT experiments with increased spatial resolution have to be performed.

In this regard, we underline that the distortions of the letters caused by the irregular and uneven surface, the holes, and the tears of the layers might hinder the identification of writing. Therefore the choice of the proper flattening procedure is essential as it can help to distinguish between the writing and the papyrus sheet and prevent data misinterpretation.

The procedures of segmentation and flattering have been performed using *ad hoc* computational programs developed by us and free software. We provide an accessible algorithm that can be reproduced, optimize, and improved.

## Materials and methods

### Samples

#### PHerc.1103

Fragment *PHerc.1103*, which dimension was 3 ×5.5 cm^2^, presents non-detachable compressed sheets. The fragment belongs to the *scorza*, the outer part of the original carbonized papyrus roll, the most exposed and, therefore, the most damaged during the eruption phases, when the papyri underwent the carbonization following exposure to high temperatures and were covered by layers of lava rock. Moreover, they had to bear very high pressure, which contributed to model the external shape, making it particularly irregular and distorted.

Before the advent of Piaggio’s machine, to reveal their content the scrolls were opened through the so-called *scorzatura* method, with which the roll was divided into two semi-cylinders. The innermost part was discarded to reach a wider surface of writing, which was copied and then scraped off to allow the lower layer to be read. The outside (the *scorza*) was the only portion that remained intact.

#### PHerc.110

According to the *Catalogo dei papiri ercolanesi*^15^, fragment *PHerc.110* belonged to a scroll partially unrolled in 1867 by Vincenzo Corazza by using Piaggio’s machine. It is inventoried along with other fragments, referred to by a single label. The fragment, mechanically removed during a manual unrolling, measures approximately $4\times 4$ cm^2^.

Both fragments are housed in *Officina dei Papiri Ercolanesi* at the National Library of Naples^15^.

### Data acquisition

The experimental data were obtained using two different X-ray imaging techniques, micro-CT and XPCT. These techniques are capable to give morphological and physical information on the inner structure of the investigated samples without the need to physically open them. Since the objects under investigation are extremely fragile it was necessary to proceed with great caution during all the steps of data acquisition because the slightest bump or rubbing could damage them.

#### X-ray microtomography

Fragment *PHerc.110* was measured using Bruker SkyScan 1172 high-resolution micro-CT scanner running at MicroX-Ray Lab of *Università degli Studi di Bari “Aldo Moro”*. The system is equipped with a polychromatic microfocus X-ray tube.

Pixel size was $12 \times 12\,\, \upmu {\mathrm{m}}^2$. To avoid physical damage to the extremely fragile object, we set the sample laying horizontally on the rotation stage inside the scan chamber.

A single scan was performed at 60 kV, 167 mA, and 2400 ms exposure time, with added filtration (0.5 Al) to improve image quality by absorbing lower-energy x-rays that tend to produce scattering. The field of view (FOV) was $50 \times 50\,\, {\mathrm{mm}}^2$ whereby a single scan was enough to image the whole sample.

#### X-ray phase contrast tomography

XPCT generates image contrast due to variation in the refractive index of the sample. It is more sensitive to density variations in light materials than conventional micro-CT and can hence lead to a deeper understanding of the analyzed object. To investigate the papyrus fragment, we have used propagation-based imaging (PBI)^16^. PBI exploits free-space propagation of a coherent or partially coherent X-ray beam to create image contrast. The wavefront, distorted by the phase-shift in the sample, propagates in some distance and gives rise to Fresnel diffraction fringes in the image on the detector.

In the experiment, carried out at ID17 of the European Synchrotron Radiation Facility (ESRF) in Grenoble, we measured *PHerc.1103* with monochromatic incident X-ray energy at 80*keV*, selected by a *Si*(111) monochromator. The sample-detector distance was set at 10 m. The detector has an effective pixel size of $47 \times 47\,\, \upmu {\mathrm{m}}^2$. FOV was limited to about 1 mm (vertical) × 75 mm (horizontal). Four vertical tomographic scans were necessary to image the whole fragment. More experimental details can be found in the publication^3^.

### Data analysis

The acquired images were flat-field and dark-field corrected.

We applied two-steps image reconstruction to XPCT experimental data: phase retrieval and tomographic reconstruction. Since in micro-CT the absorption contrast provides the direct sample projection image at the detector, only the second step was used for the micro-CT volume reconstruction.

#### Phase retrieval

There are different approaches for phase retrieval. The overview of the methods is given in Ref.^17^. Here the non-iterative algorithm developed by Paganin et al.^18^ was used. It is assumed that the object consists of a single quasi-homogeneous material with a constant ratio between real and imaginary part of the refraction index ($\delta /\beta$). Under this condition the Transport of Intensity Equation (TIE) is solved to retrieve the phase:

1 $$\begin{aligned} \phi ({\mathbf{x }}) = \dfrac{1}{2} \ln \left( FT^{-1} \left\{ \dfrac{FT \left[ I_D({\mathbf{x }})/I_0({\mathbf{x }}) \right] }{\dfrac{\beta }{\delta } + |{\mathbf{f }}|^2 \left( \dfrac{\lambda D}{4 \pi } \right) } \right\} \right) \end{aligned}$$

where *FT* and $FT^{-1}$ denote forward and inverse Fourier transformation, respectively, ${\mathbf{f }} = (f_x, f_y)$ are the Fourier coordinates, $\lambda$ is the wavelength of the radiation and $I_D({\mathbf{x }})/I_0({\mathbf{x }})$ is the normalized intensity detected at distance *D*.

#### Tomographic reconstruction

The open-source software toolkit SYRMEP Tomo Project (STP)^19^ was used to reconstruct the 3D entire volume of the sample from the XPCT projections. We applied the filtered back-projection (FBP) method^20^.

Bruker’s NRecon software^21^ was used to reconstruct the micro-CT images applying the Feldkamp-Davis-Kress (FDK) back-projection algorithm^22^.

### Surface extraction

The definition of the 3D surface starting from 3D volume is the first step in the workflow for virtual flattening. Through this step, we identify and isolate a single papyrus layer.

Due to the complicated structure and 3D arrangement of the papyrus sheets, we applied a semi-manual procedure for the papyrus sheet segmentation. For our purpose, once a suitable threshold has been chosen using global thresholding^23^, we convert the grayscale volume to binary volume, where a binary value indicates whether a voxel is associated with the material or with the empty space. The output was a binary-valued segmentation mask where we identified the boundaries of the sheet as a 3D surface. Once the surface is isolated, we re-assign to it the gray values corresponding to each voxel.

Image segmentation and surface extraction have been performed with developed in-house macros and codes using image processing program ImageJ^24^ and Python.

### Surface modelling: triangular mesh

As a next step, we approximated the surface with a triangular mesh. The process of mesh generation from a point cloud has been performed on MeshLab^25^.

At first, an array of 3D points is produced. Starting from this simple point cloud, we first evaluate the normal directions. Each point of the resulting set of 3D oriented points is associated with its 3D location and surface normal. Then the set is used as input to a modified Poisson surface reconstruction algorithm^26^. This approach formulates the surface reconstruction problem in terms of the solution of a screened Poisson equation. Some constraints are added to the equation to fit the position and normal. The algorithm is resilient to data noisy and the output is a huge triangular mesh.

### Surface unfolding: digital flattening

The transformation of a 3D surface into a 2D map is known as surface parameterization process. If the surface is a mesh, the problem is known as mesh parameterization.

Only the developable surfaces, such as plane, cone and cylinder, can be mapped from 3D to 2D without error, i.e. without metric distortions, displayed as stretching and shearing. For general (non-developable) surfaces there are various approaches to minimize different types of distortions: isometric parameterization, that has no distortion in lengths, conformal parameterization, that has no distortion in angles and equireal parameterization, without distortion in areas. Isometric parameterization is ideal but rare. In practice, conformal parameterization, equireal parameterization or some balance between them are used.

In this paper, we address the flattening problem using conformal parameterization because it is an angle-preserving method, and hence the local geometry is well retained. At the state-of-the-art, two methods are more popular than others for conformal parameterization: Least Squares Conformal Maps (LSCM)^12^ and Angle-Based Flattening (ABF)^13^. We compare both methods to understand which could be the best for our samples. We used the algorithms provided by Blender^27^.

LSCM minimizes the energy that is a measure of non-conformality of the application. This energy is invariant to arbitrary translations and rotations in the parametric space. To have a unique minimizer, it is required to fix at least two vertices. This requirement affects the results significantly. While LSCM defines a planar parameterization in terms of vertex coordinates, the ABF method defines it in terms of the angles of the planar triangles. In ABF the distortion is a function of angles and the objective is to minimize the (relative) deviation of 3D angles and 2D angles of the triangles. To ensure that the 2D angles define a valid triangulation, a set of angle constraints needs to be satisfied, i.e. that all angles are positive, the sum of angles in each triangle is $\pi$ and the sum of angles around each vertex is $2 \pi$.
